# The Role of Long Non-coding RNAs in Sepsis-Induced Cardiac Dysfunction

**DOI:** 10.3389/fcvm.2021.684348

**Published:** 2021-05-10

**Authors:** Jiawen Li, Yulin Zhang, Donghui Zhang, Yifei Li

**Affiliations:** ^1^Key Laboratory of Birth Defects and Related Diseases of Women and Children of MOE, Department of Pediatrics, West China Second University Hospital, Sichuan University, Chengdu, China; ^2^State Key Laboratory of Biocatalysis and Enzyme Engineering, School of Life Science, Hubei University, Wuhan, China

**Keywords:** long non-coding RNA, sepsis, cardiac dysfunction, biomarker, gene therapy

## Abstract

Sepsis is a syndrome with life-threatening organ dysfunction induced by a dysregulated host response to infection. The heart is one of the most commonly involved organs during sepsis, and cardiac dysfunction, which is usually indicative of an extremely poor clinical outcome, is a leading cause of death in septic cases. Despite substantial improvements in the understanding of the mechanisms that contribute to the origin and responses to sepsis, the prognosis of sepsis-induced cardiac dysfunction (SICD) remains poor and its molecular pathophysiological changes are not well-characterized. The recently discovered group of mediators known as long non-coding RNAs (lncRNAs) have presented novel insights and opportunities to explore the mechanisms and development of SICD and may provide new targets for diagnosis and therapeutic strategies. LncRNAs are RNA transcripts of more than 200 nucleotides with limited or no protein-coding potential. Evidence has rapidly accumulated from numerous studies on how lncRNAs function in associated regulatory circuits during SICD. This review outlines the direct evidence of the effect of lncRNAs on SICD based on clinical trials and animal studies. Furthermore, potential functional lncRNAs in SICD that have been identified in sepsis studies are summarized with a proven biological function in research on other cardiovascular diseases.

## Introduction

Sepsis is a syndrome with life-threatening organ dysfunction induced by a dysregulated host response to infection (1, 2). In-hospital mortality among patients with septic shock is reported to reach up to 40% (1). Septic shock is a series of circulatory, metabolic, and cellular abnormalities and is defined by a requirement for vasopressor support and persistent hyperlactatemia in the absence of hypovolemia (3, 4). Epidemiological studies showed that ~28.3 to 41% of all hospitalized sepsis patients died due to multiple organ failure (5), and sepsis-induced cardiac dysfunction (SICD) was identified as being closely associated with higher mortality rates (6, 7). Cardiac dysfunction is one of the major complications to sepsis, hence is predictive of a poor clinical outcome. Due to the pathophysiological changes of sepsis, cardiac lesions might be induced by a series of factors including myocardial ischemia, myocardial depressant substance, inflammation, adrenergic pathways deregulation, calcium overload, mitochondrial disorder, coronary microvascular dysfunction, and myocardial damages (4). Animal and cell experiments with lipopolysaccharide (LPS)-induced sepsis models demonstrated a significantly higher rate of cardiomyocyte apoptosis, intracellular ROS accumulation, elevated cytoplasm cytochrome C levels, and activated inflammatory pathways (8).

**Figure 1:**
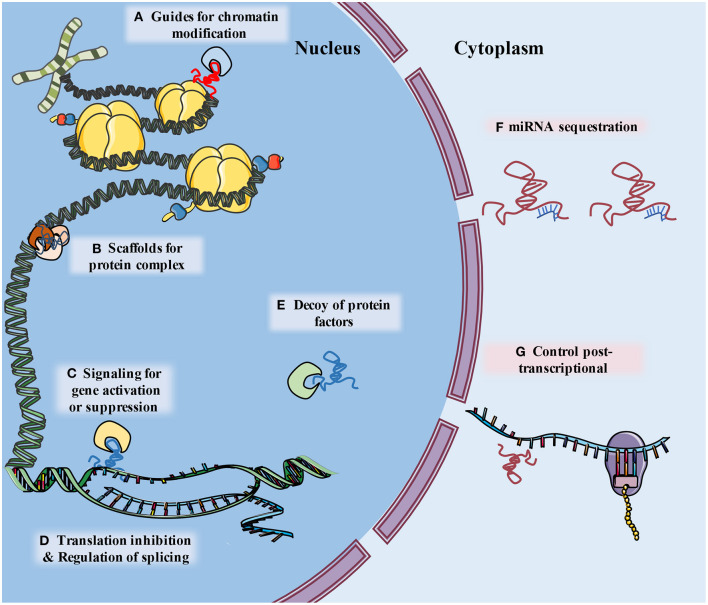
The schematic diagram describes classification of lncRNA functions. (A) LncRNAs guide ribonucleoprotein complexes to specific location of chromatin. (B) LncRNAs support assembly of protein complex. (C) lncRNAs serve as molecular signals for tissue and temporary specific activation of transcription. (D) LncRNAs can alter splicing patterns of mRNA and suppress transcription by sequestering transcription factors. (E) LncRNAs can bind to and take away protein factors, such as transcription factors and chromatin modifiers, to influence transcriptome. (F) LncRNA can “sponge” miRNA by base pairing with their complementary base sequence and reduce their effects (G) lncRNAs may interact with a variety of RNA binding proteins (RBPs), leading to alternations of mRNA stability, splicing, protein stability and subcellular localization.

The development of genome-wide association studies (GWAS) and RNA sequencing (RNA-Seq) facilitated the discovery that a large part of the nucleotide genome presents limited or no protein-coding capabilities, although these regions are still effectively transcribed. The RNAs related to these regions were named non-coding RNA (ncRNA) (9). Long non-coding RNA (lncRNA) is a type of ncRNA that is composed of more than 200 nucleotides and contributes to transcriptional and post-transcriptional regulation of RNA. According to their molecular function, lncRNAs can be classified as signal, decoy, guide, scaffold, enhancer, or sponge lncRNAs (especially circular RNAs) (10, 11) (Figure 1). Whether circular RNAs (circRNAs) belong to the lncRNAs is a matter of controversy. However, in consideration of their similarities in function and definition to lncRNAs, we regard circRNAs as a unique subtype of lncRNA, and consequently they are included in this review (11–13).

Modulation of lncRNA plays important roles in various stages of sepsis development and pathophysiological processes, and this may offer potential novel diagnostic and therapeutic strategies to reduce the mortality and burden of SICD. Using sequencing analysis, more than 80% of the primary genetic elements were observed to change in patients with critical sepsis (14). *In vitro*, human umbilical vein endothelial cells (HUVECs) exposed to LPS showed a 28- to 70-fold increase in the expression of lncRNAs (15). Differential expression of lncRNAs has been observed in several other cell types after exposure to the plasma of septic patients or LPS, including human tubular epithelial cells, monocytes, and cardiomyocytes, indicating a tissue-specific biological function of lncRNA (16–18). Therefore, the lncRNAs involved in SICD regulate both cardiomyocytes and non-cardiomyocytes. Current evidence indicates a role for lncRNAs in regulation of cardiomyocyte functions, such as mitochondrial homeostasis, calcium handling, contraction, and apoptosis.

Activation of inflammatory pathways mediated by Toll-like receptor (TLR) signaling in response to pathogen-associated molecular patterns (PAMPs) and damage-associated molecular patterns (DAMPs) is an important mechanism of cardiomyocyte injuries caused by sepsis. These inflammatory pathways include those involving nuclear factor-κB (NF-κB) and mitogen-activated protein kinase (MAPK), as well as some other pathways (19). The lncRNAs involved in immune responses are also likely to contribute to the origins of SICD. However, since lncRNAs present multiple modalities of action with low conservation in vertebrates, exploring the individual functions of a particular lncRNA is challenging and more difficult than similar research on microRNAs (miRNAs). Hence, several lncRNAs involved in inflammatory responses in cardiomyocytes lack associated evidence in SICD (20).

This review summarizes the direct evidence for the involvement of lncRNAs in SICD based on clinical research studies of patients with SICD and basic biology explorations using animal or cell models of SICD. Furthermore, the lncRNAs involved in both sepsis and cardiovascular diseases (CVD) among individual studies are described and their potential associations in SICD are analyzed; these studies were treated as indirect evidence for the role of lncRNAs in SICD.

## The Association Between lncRNAs and SICD

A recent study using microarray and whole genomic transcription sequencing with bioinformatics analyses on blood samples from patients with sepsis discovered 46 differentially expressed lncRNAs (DElncRNAs) (21). Additionally, 28 upregulated and 61 downregulated lncRNAs were identified in the public reported NCBI GEO dataset (22). Similar analyses based on cardiac tissue from mouse or rat sepsis models reported 74 (23) to 1,275 DElncRNAs, and revealed 14 lncRNAs that were highly correlated with 11 mitochondria-related differentially expressed mRNAs (24) and 11 differentially expressed circRNAs (25). Kyoto Encyclopedia of Genes and Genomes (KEGG) analysis indicated that upregulated lncRNAs were significantly enriched in the p53, NF-κB, and HIF-1 signaling pathways (26). Tissue-specific RNA-Seq in artificial induced inflammation revealed that some LPS-mediated lncRNAs were correlated to cardiometabolic traits (16). Thus, lncRNAs participate in regulating mitochondrial function, metabolic homeostasis, and inflammation signaling in cardiomyocytes during sepsis attacks.

Evidence in the literature linking lncRNAs and SICD can be divided into two distinct types. The first type of evidence (direct) presents clear confirmation of the involvement of lncRNAs in SICD, either from clinical samples or animal models, with definite molecular function demonstrated. The second type of evidence (indirect) describes studies where lncRNAs displayed differentiated expression in sepsis samples and were proven to have a critical role in maintaining cardiomyocyte function but lacked convincing evidence in SICD.

## lncRNA Involved in SICD Among Various Cell Types

**Figure 2:**
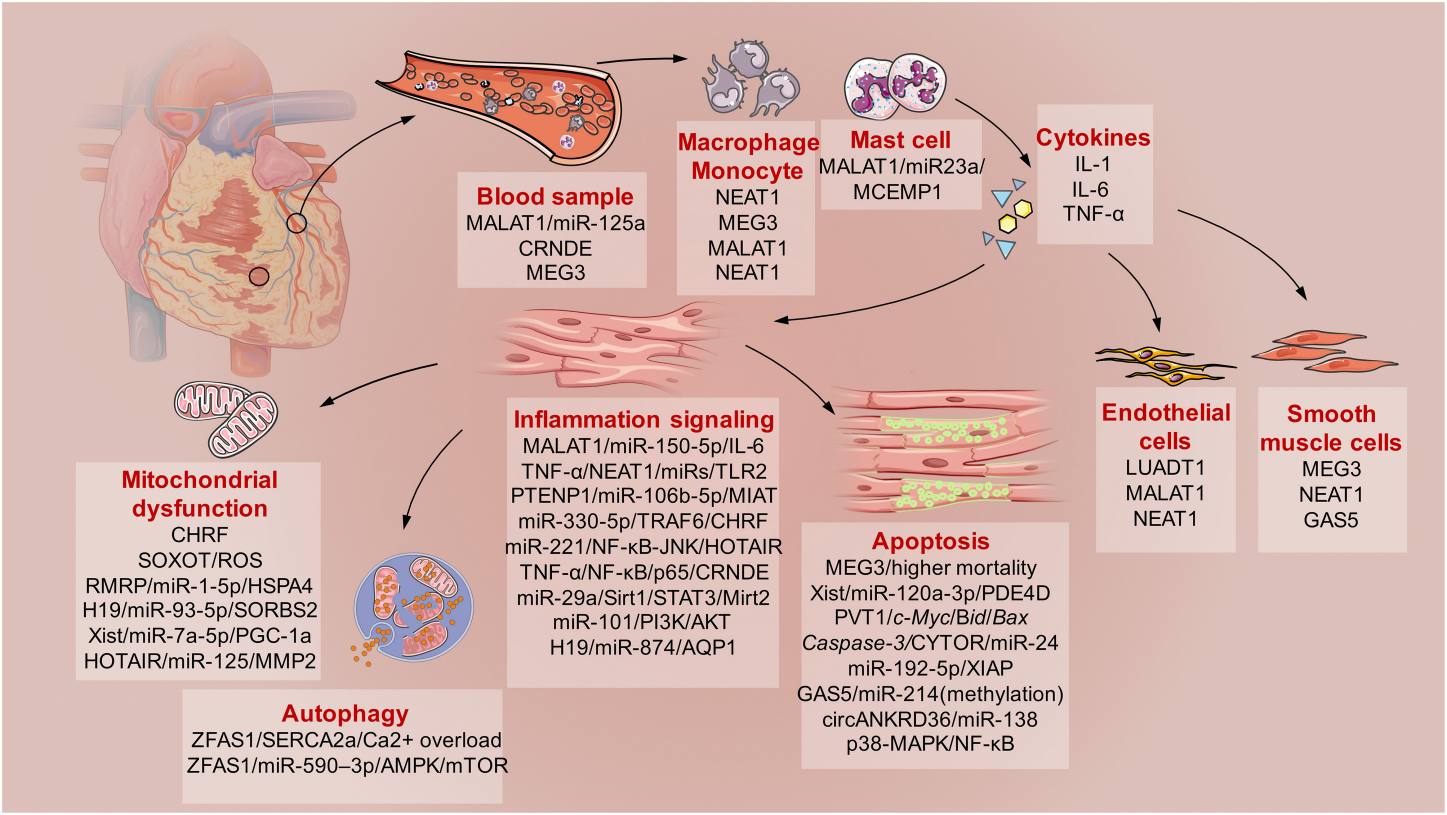
The schematic diagram describes the involved lncRNAs in sepsis induced cardiac dysfunction (SICD). Generally, current evidences demonstrated some lncRNAs served as biomarkers for SCID. Then, monocytes, macrophage, and mast cells would be activated with kinds of cytokines secretion. After that, immune responses of cardiomyocytes would lead to mitochondrial dysfunction, apoptosis and autophagy under the regulation of specific lncRNAs. Besides, lncRNAs also participates in the regulation of endothelial cells and smooth muscle cells during SICD.

Here, direct evidence of the involvement of lncRNAs in SICD is summarized (Figure 2). This evidence is based on the findings from basic molecular biological research using animal models of hypodynamic septic shock induced by LPS and cecal ligation and puncture (CLP) (27); cardiac muscle cell lines (primary culture cardiomyocytes, H9C2, HL-1, and AC-16 cell lines) and microvascular cell lines exposed to serum from septic patients or administered with LPS (28); and clinical studies of sepsis patients subjected to cardiac dysfunction (Table 1).

**Table 1:** Direct evidence of lncRNAs in SICD.

**LncRNA**	**Bindings**	**Downstream factors**	**Molecular function**	**Sepsis Models (*in vitro* + *in vivo*)**	**Outcomes**
MALAT1↑	miR-150-5p↓	–	miRNA sponge	H9C2 + LPS	IL-6↑ TNF-α↑ NF-κB signaling pathway↑
↑§	–	SAA3↑	–	HL-1 + LPS Mice + LPS	TNF-α↑
↑	miR-125b↓^*^	–	–	H9c2 + LPS Rat +CLP	p38 MAPK/ NF-κB↑
NEAT1↓φ	miRNAs of inflammatory indicators	TLR2 and p-p65↓	–	Mice + LPS	Myocardial Pathological ↓ Injury Myocardial Apoptosis↓ Oxidative Stress↓ Inflammation↓ TLR2/NF-κB signaling pathway↓
↓	miR-144-3p↓	p-IκBα and p-p65↓	miRNA sponge	HL-1 + LPS	Myocardial Cell Injury↓ NF-κB Signaling Pathway↓
PTENP1↓	miR-106b-5p↑	–	miRNA sponge	H9c2 + LPS Mice + CLP	Cell viability↑ IL-6↓and TNF-α↓ Inflammation↓
MIAT↑	miR-330-5p↓	TRAF6↑	miRNA sponge	HL-1 cells + LPS Mice + LPS	TRAF6/NF-κB signaling axis↑
CHRF↓	miR-221↑	P65↓	miRNA sponge Protein localization	H9c2 + LPS	Mitochondrial apoptosis↓ Cell viability↓ Apoptosis rate↓ IL-6 and TNF-α↓ NF-κB↓ and JNK pathways↓
circANKRD36z↓	miR-138↑	–	miRNA sponge	H9c2 + LPS	Apoptosis↓ and inflammatory injury↓ p38MAPK/NF-κB↓
HOTAIR↑$	–	–	–	HL-1 + LPS Mice + LPS	TNF-α↑ phosphorylation of NF-κB p65 subunit↑
CRNDE↑§	miR-29a↓	SIRT1↑	miRNA sponge	H9c2 + LPS Rat + LPS	Cardiomyocyte apoptosis↓ Oxidative stress↓ phosphorylated NF-κB p65↓ and Cleaved PARP1↓ NF- κB/PARP1 signaling↓
Mirt2↑	miR-101↓	–	miRNA sponge	Rat + CLP	IL-1β↓, IL-6↓, TNF-α↓, MPO↓ IL-10↑ PI3K/AKT Signaling Pathway↓
rPVT1↓φ	Irak-2↓	c-Myc↓ Myd88↑	Protein stabilization	H9C2 + LPS	Myocardial Depression↑ Cell Apoptosis↑
Xist↓	mir-7a-5p#	PGC-1α↑ Tfam↓	–	Mouse cardiomyocytes MCM cells + LPS	Cardiomyocyte ATP levels↑ Cardiomyocyte apoptosis↓
CYTOR↑	miR-24↓	XIAP↑	miRNA sponge	H9c2 + LPS Mice + LPS	viability↑ Apoptotic↓ TNF-α↓and IL-1β↓ LDH↓
KCNQ1OT1↑	miR-192-5p↓	XIAP↑	miRNA sponge	H9c2 + LPS Rat + LPS	Proliferation↑ Apoptosis↓ TNF-a↓, IL-1b↓, and IL-6↓
CircHIPK3↓	–	–	miRNA sponge?	H9c2 + LPSMice + CLP	Heart damage markers↓ And myocardial apoptosis↓ Oxidative stress↓ and Inflammation↓
MEG3↓	P53‡	–	–	AC16 + LPS Plasma from sepsis patients	Apoptosis↓
GAS5↑φ	miR-124↓	–	miRNA Methylation	AC16+LPS	Apoptosis↓
H19↓	miR-93-5p↑	SORBS2↓	miRNA sponge	H9C2 + LPS Sepsis patients	Cell growth inhibition↑ Mitochondrial damage↑
↓	miR-874↑	AQP1↓	miRNA sponge	UL-1 + LPS Serum from peripheral blood samples of sepsis patients	TNF-α, IL-6, and IL-1β↑
CHRF↓	miR-221↑	P65↓	miRNA sponge Protein localization	H9c2 + LPS	Mitochondrial apoptosis↓cell viability↓apoptosis rate↓ IL-6 and TNF-α↓ NF-κB↓ and JNK pathways↓
RMRP↑	miR-1-5p↓	HSPA4↑	miRNA sponge	HL-1 + LPS Mice + LPS	Apoptosis↓ MMP↑ Mitochondrial damage↓
SOX2OT↓$	SOX2↑	–	Transcriptional suppression	H9c2 + LPS Mice + LPS	MMP↑ Mitochondrial reactive oxygen species↓ Mitochondrial dysfunction↓
ZSAF1↓$	miR-590–3p↑	–	Base paring	Mice + CLP	Pyroptosis↓ Autophagy↑ AMPK/mTOR signaling↓
MALAT1↓∧	EZH2	EZH2	Histone modification	CMVECs isolated from rats + LPS Rat +CLP	CMVEC cell hyperpermeability and apoptosis ↓
LUAD1↑	miR-195†	Pim-1↑	Base paring	Plasma from sepsis patients HCAECs	Apoptosis of HCAECs↓

*Downstream factors included proteins which are reported to be directly modulated by lncRNAs or their binding molecules and gene locus*.

*Rising arrow or a falling arrows of lncRNAs depend on the regulation of included studies, not on their expression change after sepsis. Direction of arrow of downstream factors and outcome relies on direction of arrows of lncRNAs*.

* *MiR-125b was proved to modulate MALAT1 as a upstream regulator*.

∧ *MALAT1 was downregulated by ulinastatin*.

& *PVT1 upregulates Myd88 by protein stabilization but it's unknown how PVT1 downregulate c-Myc*.

# *Database analyses found that Xist has a binding site of miR-7a-5p, but there is no direct modulatory relationship between these two non-coding RNAs*.

$ *Evidences of studies were acquired based on transgenic mouse*.

§ *Researchers of included studies screened lncRNAs by microarray*.

φ *Researchers of included studies screened lncRNAs by RNA-sequencing*.

† *LUADT1 and miR-195 demonstrate strong base paring between each other, but overexpression of LUADT1 and miR-195 did not significantly alter the expression of each other*.

‡ *lncRNA MEG3 may interact with p53 to regulate cancer cell apoptosis and it may be involved in the pathogenesis of sepsis by a similar mechanism*.

? *Included study did not mention mechanism of this lncRNA, but other study reported its mechanism*.

### Cardiomyocytes

LncRNAs participate in cardiomyocyte function through inflammatory signaling pathways, cytokine release, mitochondria homeostasis, apoptotic processes, and cell proliferation and migration during SICD.

#### Inflammation Signaling

LncRNAs are involved in the inflammatory process by regulating inflammation signaling, including the NF-κB, JAK/STAT, and MAPK pathways, and production of cytokines, such as IL-1, IL-6, IL-10, and TNF-α. The lncRNA MALAT1 is responsible for the septic inflammatory response under LPS administration in cardiomyocytes by downregulating miR-150-5p to increase expression of IL-6, TNF-α and the NF-κB signaling pathway (29), and TNF-α induction partly relied on serum amyloid antigen 3 (SAA3) (30). MALAT1 also interacts with p38 MAPK/NF-kB and miR-125b to aggravate cardiac inflammation and dysfunction in sepsis (31). The lncRNA NEAT1 was associated with disease severity, higher mortality risk, and unfavorable prognosis in sepsis patients (32). Furthermore, NEAT1 plays an important role in cardiomyocyte injury and apoptosis associated with miR-140-5p, miR-193a, miR-27b, miR-181b, miR-129-5p, miR-495-3p, miR-125a-5p, and their corresponding downstream regulated genes (33–40). NEAT1 knockdown can improve the outcome of LPS-induced myocardial injuries in mice by upregulating miR-144-3p (41) and downregulating expression of TLR2 and p65 and mRNA levels of inflammatory indicators to inhibit the TLR2/NF-κB signaling pathway (42). Expression of lncRNA PTENP1 was upregulated in sepsis models subjected to LPS administration, while miR-106b-5p expression was downregulated. Matrine administration could attenuate changes in expression of these two ncRNAs, and the cardioprotective effects of matrine were reversed by overexpression of PTENP1 or knockdown of miR-106b-5p (43). The lncRNA MIAT directly binds to miR-330-5p to activate TRAF6/NF-κB signaling axis and further promotes inflammatory response as well as oxidative stress in LPS-induced septic cardiomyopathy (44).

Silencing the lncRNA CHRF protected H9c2 cells against LPS-induced injury via upregulation of miR-221 and modulation of NF-κB and JNK pathways (45). In addition, silencing HOTAIR lncRNA reduced secretion of TNF-α into the circulation by inhibition of NF-κB signaling through dephosphorization of NF-κB p65 subunit, and helped preserve cardiac function in septic mice (18). Moreover, knockdown of circHIPK3 effectively alleviated LPS-induced myocarditis (46).

Beyond the lncRNAs that contribute to triggering inflammation, there is a series of lncRNAs that present a protective value of SICD. LncRNA CRNDE attenuates miRNA-29a to enhance expression of *Sirt1*, which contributes to inhibition of NF-κB and STAT3 inflammation signaling in myocardial tissue under septic attack (47). LncRNA Mirt2 silenced miR-101 and attenuated the myocardial inflammatory response in sepsis rats through the PI3K/AKT signaling pathway, and this improved cardiac remodeling and function (48). However, no human homologs of Mirt1 and Mirt2 have been described to date.

In an *in vitro* model established on cardiomyocytes subjected to LPS, there was a negative relationship between lncRNA H19 and miR-874, and a positive correlation between H19 and Aquaporin 1 (AQP1). H19 could act as AQP1 competing endogenous RNA (ceRNA) by regulating miR-874 and restoring LPS over-activated inflammatory responses and myocardial dysfunction (49, 50).

#### Mitochondria

Mitochondria are one of the most important organelles of cardiomyocytes, but they are quite sensitive to external and internal stimulations, resulting in mitochondrial dysfunction and leading to metabolic disorder with accumulation of reactive oxygen species (ROS). Mitochondrial dysfunction is associated with DNA damage and apoptosis. In experimental models of sepsis attacks, reduced mitochondrial membrane potential (MMP), elevated mitochondrial cytochrome C, and downregulated ROS scavenging were identified (8). LncRNAs make significant contributions to maintaining cardiac mitochondria homeostasis, hence these studies revealed a critical role of lncRNAs in response to sepsis attack.

Zhang et al. (45) demonstrated that silencing the lncRNA CHRF prevented LPS-triggered mitochondrial apoptosis and inflammation of cardiomyocytes. LncRNA SOX2 overlapping transcript (SOX2OT) is a proven mitochondrial damage factor in sepsis and contributes to mitochondrial dysfunction progression by inhibiting SOX2 expression in septic cardiomyopathy. Knockdown of SOX2OT could restore the MMP, along with reduction of ROS production induced by LPS, while overexpression of SOX2OT enhanced mitochondrial damage (51).

LncRNA RMRP acts as a sponge for miR-1-5p and provides a protective effect to mitochondria via the RMRP-miR-1-5p-HSPA4 network, which is known to play crucial roles in inflammation (8). LncRNA H19 and SORBS2 (Sorbin and SH3 domain-containing protein 2) were downregulated in H9C2 cells following administration of LPS, and miR-93-5p was simultaneously upregulated. LncRNA Xist is instrumental in X-chromosome inactivation and inhibits apoptosis in acute myocardial infarction (MI) (52). Peroxisome proliferator-activated receptor-γ coactivator-1α (PGC-1α) and adenosine triphosphate (ATP) expression was markedly reduced in sepsis leading to mitochondrial dysfunction, but mitochondrial function was restored after the inhibition of Xist and mir-7a-5p, which reduced apoptosis in response to LPS (53). Inhibition of lnc-HOTAIR aggravates oxidative stress-induced damage of H9c2 cells through the HOTAIR/miR-125/MMP2 axis (54).

LncRNA H19 is an important regulator of mammalian development and disease in that it inhibits cell proliferation (55). H19 is normally highly expressed during *in utero* development and then downregulated at birth (56), while re-expression occurs in some cardiovascular disease settings (57–59). In accordance with its inhibition function of cell proliferation, H19 was proved as precursor of miR-675, which inhibits cardiomyocyte hypertrophy and contributes to cardiac fibroblast proliferation and fibrosis through repression of DUSP5/ERK1/2 (60). Furthermore, H19 is involved in myocardial ischemic preconditioning via increasing the stability of nucleolin protein, which mitigates the damage caused by MI (61). Human GWASs demonstrated significant associations between the H19 locus and systolic or mean arterial blood pressure (9). LPS-induced cell growth inhibition and mitochondrial damage was significantly reversed by overexpression of H19, which sponged miR-93-5p to promote SORBS2 expression (62).

#### Apoptosis

Cardiomyocyte apoptosis, which is a key parameter for SICD and leads to long-term myocardial dysfunction, has been proposed to occur as a result of a sequence of cellular damages (63). Several signaling pathways are involved in apoptosis regulation via nuclear and mitochondrial approaches. However, crosstalk between lncRNAs and signaling pathways has been identified, and several lncRNAs regulate the process of apoptosis.

Overexpression of lncRNA MEG3 is associated with high mortality rates in patients with sepsis, thus is indicative of poor clinical outcomes and is believed to be associated with LPS-induced renal epithelial cell and cardiomyocyte apoptosis (64). LncRNA Xist promoted apoptosis of cardiomyocytes and inhibited proliferation of these cells by downregulating miR-130a-3p and upregulating *PDE4D*, which is a direct target of miR-130a-3p (52).

LncRNA PVT1 also showed significant upregulation and a vital functional role in maintaining the myocardial contractile function in rat models of hypodynamic septic shock induced by LPS. Knockdown of PVT1 induced cell apoptosis in LPS-induced cardiomyocytes through increasing the expression of *c-Myc, Bid, Bax*, and *Caspase-3* and decreasing expression of *Myd88* and *Bcl-2* (23). LncRNA CYTOR was markedly downregulated during sepsis. This lncRNA negatively regulated expression of miR-24 and apoptosis-related proteins that were regulated by miR-24. MiR-24 directly targeted the 3′UTR of X-chromosome-linked inhibitor of apoptosis (XIAP) and suppressed its expression. Downregulation of CYTOR aggravated sepsis-induced cardiac injury via regulation of miR-24/XIAP (65). The lncRNA KCNQ1OT1 is similar in mechanism to CYTOR. It was considerably downregulated in myocardial tissues of septic rats, whereas miR-192-5p was increased in these tissues. CYTOR regulates XIAP through miR-192-5p, which pairs with the 3′UTR of XIAP, and represses its protein translation. These findings show that downregulation of KCNQ1OT1 aggravates cardiac injury through the miR-192-5p/XIAP axis during sepsis (66). LncRNA GAS5 may upregulate miR-214 through a methylation pathway to inhibit cardiomyocyte apoptosis in sepsis (67).

Fan et al. (46) demonstrated that circHIPK3 expression was significantly upregulated when exposed to LPS *in vivo* and *in vitro*, and that knockdown of circHIPK3 effectively alleviated LPS-induced myocarditis by attenuating inflammation-induced apoptosis of cardiomyocytes. Furthermore, silencing circANKRD36 exerted an anti-inflammatory and anti-apoptosis function in LPS-exposed H9c2 cells via the p38-MAPK/NF-κB pathway and upregulation of miR-138 (68). Another study confirmed an association between circANKRD36 and miR-15/MyD in regulating apoptosis due to inflammation damage (69).

#### Autophagy

Autophagy is an important biological process for regulating cellular homeostasis. However, there is currently limited data demonstrating the involvement of lncRNAs in regulating cardiomyocyte autophagy. One study revealed that lncRNA ZFAS1 was an endogenous SERCA2a inhibitor and induces mitochondria-mediated apoptosis via cytosolic Ca2+ overload (70). ZFAS1 is activated by the transcription factor SP1 and aggravates the progression of sepsis-induced cardiac dysfunction via miR-590–3p/AMPK/mTOR signaling-mediated autophagy and pyroptosis of cardiomyocytes (71, 72).

### Immune Cells

During sepsis, the immune system is the frontier responding to harmful stimulations, and monocytes, macrophages, and neutrophils all make significant contributions to targeting organ damage. The innate immune response induces strong activation of the cytokine system, which has plethoric effects on various organs and the vasculature, leading to changes in vascular permeability, endothelial function, and activation of further mediators such as bradykinin, histamine, and the complement and coagulation systems.

The lnc-MALAT1/miR-125a axis presents excellent value in differentiating sepsis patients from healthy controls using peripheral blood samples (73). In another study using clinical blood samples, lnc-CRNDE was found to trigger inflammation through the TLR3-NF-κB-cytokine signaling pathway and the downstream release of inflammatory cytokines (74). As a major protein related to innate immune and inflammatory responses, TLR3 is known to cause cardiac dysfunction and other organ damage during sepsis (74, 75). Low expression of lnc-MEG3 might also serve as a potential biomarker for the development, progression, and prognosis prediction of sepsis (76–78). Furthermore, overexpression of MEG3 prevented LPS-induced macrophage apoptosis and secretion of inflammatory factors by inhibiting activation of the NF-κB signaling pathway (77).

Lnc-MALAT1 plays multiple roles in inflammatory stimulation in the macrophage cell line RAW264.7 (79). This lncRNA could inhibit the proliferation of LPS-stimulated RAW264.7 cells by inducing SMAD3 expression via downregulation of hsa-miR-346 (79). Lnc-MALAT1 also promotes inflammation in septic mice by binding to miR-23a to upregulate mast cell-expressed membrane protein 1 (MCEMP1) (80).

High expression of NEAT1 in peripheral blood mononuclear cells (PBMCs) can be considered as an additive marker for the diagnosis of sepsis (81), while another study confirmed that monocyte-enriched NEAT1 was suppressed in post-MI patients (82). Data from experiments with NEAT1-knockout (NEAT1-KO) mice identified NEAT1 as a novel lncRNA-type immunoregulator affecting monocyte-macrophage functions and T cell differentiation. NEAT1-KO marrow-derived macrophages (BMDMs) responded to LPS with increased ROS production and disturbed phagocytic activity (82).

### Endothelial Cells

Cardiomyocytes are the dominant type of cells in the heart. However, various cell types comprise functional heart tissue. Endothelial cells contribute to form microvascular circulation in myocardia. Endothelial cell dysfunction impairs the micro-circulation function, inducing ischemic cardiac lesions. In sepsis attacks, endothelial cells are also major targeted sites. However, few studies have drawn correlations between lncRNAs and endothelial cell damage. The lncRNA LUADT1 was downregulated in patients with sepsis and in cultured human primary coronary artery endothelial cells (HCAECs) exposed to LPS. Overexpression of LUADT1 upregulated the expression of *PIM1*, a target of miR-195. These findings indicated that overexpression of either LUADT1 or *PIM1* would reduce the damage effects of miR-195 on LPS-induced apoptosis of cardiac endothelial cells (83). Yu et al. (84) demonstrated that the drug Ulinastatin protected against LPS-induced cell hyperpermeability and apoptosis of cardiac microvascular endothelial cell (CMVECs) via downregulation of lncRNA MALAT1 and EZH2. Moreover, Liu et al. (85) reported that miR-150 could induce sepsis-induced endothelial injury by regulating endoplasmic reticulum (ER) stress and inflammation via the MALAT1-mediated NF-κB pathway. Lnc-NEAT1 also participates in the viability and survival of coronary endothelial cells (86, 87).

### Smooth Muscle Cells

Smooth muscle cells also significantly contribute to maintenance of coronary vessel circulation. However, smooth muscle cells were the targets of inflammation damage due to sepsis attacks. Ahmed et al. (88) demonstrated a role of NEAT1 in regulating phenotypic switching by repressing smooth muscle-contractile gene expression through an epigenetic regulatory mechanism. Silencing lnc-NEAT1 in vascular smooth muscle cells (VSMCs) enhanced expression of smooth muscle-specific genes while attenuating proliferation and migration of the VSMCs. The lncRNA MEG3 could modulate the balance of proliferation/apoptosis in VSMCs by regulating the miR-26a/SMAD1 axis (89). In addition, the lncRNA GAS5 exacerbates hypertensive arterial remodeling by regulating VSMC phenotypic conversion, which leads to microvascular dysfunction (90). However, there is lacking convinced evidence of GAS5 on SICD.

## Predicted lncRNAs Based on Available Evidence

In addition to the above-mentioned lncRNAs with direct evidence in SICD, some other lncRNAs were reported to be involved both in sepsis and some types of CVD by other mechanisms. In view of the molecular functions of lncRNAs in regulating cardiomyocyte homeostasis and their expression during sepsis but without convincing evidence presented in a single study focusing on SICD, the most reported lncRNAs and associated mechanisms are summarized in this review to demonstrate their comprehensive impacts. Table 2 lists the lncRNAs that we predicted might play a role in SICD although no direct evidence is available from biological experiments or clinical trials. These lncRNAs were found to express differentially or function in sepsis and participate in CVD or other cardiac psychopathological processes in individual studies.

**Table 2:** Summary of potential lncRNAs in SICD based on available evidence.

**LncRNA**	**Disease**	**Expression**	**Samples or tissue/cell source**	**Downstream factors**	**Molecular function**	**Function**
ANRIL	Sepsis	Up	Plasma from patients of sepsis	miR-125a↓	–	Biomarker of severity, inflammation, and prognosis
	AMI	Up	Mice myocardial tissue HL-1	Deubiquitinase USP17 IL-33 ST2	–	Apoptosis↑
	MI	Up	Ischemic hearts HUVECs	Akt phosphorylation↑	–	Cell migrations↑ and Tubulogenesis↑ Ischemia-induced Angiogenesis↑
	Inflammation-relevant CAD	Up	CAD patients HCAECs HUVECs CAD mice	miR-181b↓ EMT-specific Proteins	–	Inflammatory factors↓ and Vascular-protective factors↓
UCA1	Sepsis	Up	HMECs	–	–	Pre-inflammatory mediators↑
		Up	WI-38 cells	miR-499b-5p↓ TLR4↓	decoy	Inflammatory injury ↑apoptosis↑
	I/R§	Up	H9C2 cells	–	–	ER stress↓ and Cell apoptosis↓ Mitochondria Dysfunction↓ and Oxidative stress↓
Lnc-DC	Sepsis	Up	kidneys and liver	Stat3↑ Toll-Like Receptor 4↑	–	Pro-inflammatory factors↑
	CAD	Up	PBMCs	STAT3↑	–	JAK/STAT pathway↑
THRIL	Sepsis	Up	Blood extraction from sepsis patients HBEpCs	miR-19a↓	miRNA sponge	TNF-α↑
	CAD	Up	CAD blood samples EPC	FUS	Protein binding	Cell viability↓ cell autophagy↑ Cell proliferation↓ AKT pathway↑
	MI	Up	H9C2	miR-99a↓ Brg1↓	miRNA sponge	Cell injuries↑ PI3K/AKT and mTOR Signaling pathways↓
HULC	Sepsis	Up	HMECs	–	–	Pre-inflammatory mediators↑
	TNF-α↑	Down	HUVECs	miR-9↓	DNA methyltransferases	Apoptosis↓
	I/Rφ	Down	Rat myocardial tissue H9C2	miR- 377-5p↓	miRNA sponge	Cardiomyocyte apoptosis↓
Lnc-P21	Sepsis^*^	Up	–	–	–	Macrophage activation Septic shock susceptibility autophagy Cardiomyocyte adherens junctions
	CAD	Down	HA-VSMC RAW264.7 Carotid arteries	MDM2↑	Enhancer	Cell proliferation↓ Apoptosis↑ Neointima Formation↓
TUG1	Sepsis	Down	Serum samples from urosepsis patients RMC	miR-142-3p↓ sirtuin 1↑	miRNA sponge	Cell viability↑ Apoptosis↓ Cytokines production↓ Autophagy↓
	Hypertension	Up	Rat isolated VSMCs	miR-145-5p↓ FGF10↑	miRNA sponge	Proliferation↑ Migration of VSMCs↑
	Atherosclerosis	Up	RAW264.7 MOVAS Mice aorta and aortic sinuses	miR-133a ↓ FGF1↑	miRNA sponge	Cell growth↑ Inflammation↑ Apoptosis↓
SNHG16	Sepsis§	Down	Blood sample from sepsis or respiratory infection/pneumonia RAW264.7	miR-15a/16↓ TLR4↑	binding miRNAs	Inflammatory pathway↓
	CAD	Up	Peripheral blood from sepsis patients HCASMC	miR-218-5p↓	Decoy	Proliferation and migration of HCASMC cells↑ Apoptosis↓
	Cardiac hypertrophy	Up	H9c2	miR-182-5p↓ IGF1↑	miRNA sponge	Cardiac hypertrophy↑
aHIF	Sepsis^*^	–	–	–	–	Expression Profiling Golgi stress Acute lung injury
	End-stage heart failure	Up	Human heart tissues	HIF↓	Antisense transcript	–
	MI	Up	Peripheral blood cells	–	–	–

* *These lncRNAs are involved in pathophysiologic process of sepsis or CVD but there is no direct evidence involved in SICD*.

§ *Researchers of included studies screened lncRNAs by microarray*.

φ *Researchers of included studies screened lncRNAs by RNA-sequencing*.

*The direction of arrow of downstream factors indicates function of lncRNAs, not their change in status of diseases*.

*The direction of arrow of function indicates outcome of restored or upregulated lncRNAs*.

### LncRNAs That Present Similar Functions in Sepsis and CVD

The mechanisms of lncRNAs in regulating downstream signaling is complicated, although research on ncRNAs is growing. However, direct evidence of lncRNAs on SICD remain limited. Moreover, sepsis is considered as a type of syndrome that damages various organs. Hence, research on sepsis includes investigations on various damages beyond cardiac dysfunction, such as lung injuries, kidney disorders, and other damages. Based on this predicament, we selected to review the lncRNAs involved in sepsis without evidence based on SICD but which had been confirmed as having similar protective or adverse roles in other types of CVD. The lncRNAs outlined in this part of the review are highly likely to have their capabilities proven in future SICD studies.

#### ANRIL

Several studies demonstrated that the lnc-ANRIL/miR-125a axis could serve as a predictor for prognosis, severity, and inflammation among sepsis patients (91–93). LncRNA ANRIL is the prime candidate gene at Chr9p21 and widely recognized as a critical part of endothelial inflammation and cell proliferation (91, 94–97). Single nucleotide polymorphisms (SNPs) and splice variants of ANRIL were reported to regulate endothelial cell activities involved in coronary artery heart disease (CAD) and MI (98–103). Abnormal expression of ANRIL is associated with vascular endothelium injury and proliferation, migration, and apoptosis of VSMCs; which also contribute to mononuclear cell adhesion and proliferation (104, 105). ANRIL knockdown induced cardiomyocyte apoptosis in acute MI by regulating IL-33/ST2 or Akt (106, 107). Enhanced expression of ANRIL and suppressed expression of miR-181b, which was inhibited by ANRIL, were recorded in CAD populations and confirmed ANRIL as an independent risk factor (108).

#### DC

Lnc-DC, also known as whey acidic protein/four-disulfide core domain 21 (Wfdc21), was reported to be correlated with immune responses. Knockdown of lnc-DC downregulated expression of pro-inflammatory factors, such as IL-1β and TNF-α, in LPS-treated macrophages through the STAT3/TLR4 signaling pathway (109). Alikhah et al. (110) found significant correlations between expression of lnc-DC with SOCS1 and STAT3 in CAD patients.

#### THRIL

LncRNA THRIL is upregulated during sepsis and may serve as a sponge of miR-19a to upregulate TNF-α (111). This lncRNA is considered to play important roles in the innate immune response and inflammatory diseases in humans (112). THRIL mediates autophagy of endothelial progenitor cells via the AKT pathway and FUS (113). Knockdown of THRIL protected H9C2 cells against hypoxia-induced injuries by regulating miR-99a (114). This mechanism was further demonstrated by Sheng et al. (115) with the observation that Geniposide alleviated hypoxia-induced injury through downregulation of THRIL in H9c2 cells. In addition, THRIL was increased in CAD patients and proved as a biomarker to evaluate CAD risk (116).

#### SNHG16

The lncRNA SNHG16 can act as a ceRNA to downregulate the miR-15a/16 cluster, reducing LPS-induced inflammatory signaling (117). SNHG16 also helps regulate miR-218-5p and promotes the proliferation and migration of coronary artery VSMCs via the Wnt/β-catenin pathway, protecting against the injuries from MI (118). Furthermore, silencing of SNHG16 repressed Ang II-imposed cardiac hypertrophy by targeting the miR-182-5p/IGF1 axis (119).

### lncRNAs That Present Opposite Roles in Sepsis and CVD

Selection of potential lncRNAs involved in SICD is difficult, even with meticulous attention. Some findings from different individual studies demonstrated opposing functions of lncRNAs between sepsis and CVD, either in a protective or adverse direction. However, it is possible that there may be a shared intermediate target. Here, such lncRNAs are briefly described, but further analysis is required in relation to these lncRNAs and SICD. Moreover, the long-term effects of lncRNAs on CVD also lacks convincing data and this is another area that requires further research.

#### UCA1

Upregulation of lncRNA UCA1 is necessary for the response of pro-inflammatory immune cells during LPS-induced sepsis (120, 121). However, UCA1 inhibits ischemia/reperfusion (I/R)-induced oxidative stress and mitochondria dysfunction via suppression of ER stress (122).

#### HULC

HULC could induce pro-inflammatory mediators in response to LPS exposure in endothelial cells (120). Overexpression of HULC in HUVECs promoted angiogenesis by increasing cell viability, proliferation, and tube-like structure formation through downregulation of miR-29b (123). HULC also participated in TNF-α- (124) and I/R- (125) induced cardiomyocyte apoptosis through regulation of miR-9 and miR-377-5p expression.

#### P21

LncRNA-p21 serves as a repressor in p53-dependent transcriptional responses (126). This lncRNA regulates neointima formation, VSMC apoptosis, and atherosclerosis by enhancing p53 activity (127). Expression of lncRNA-p21 was significantly increased in a septic model and it predominantly functioned in *cis* to activate expression of p21, its neighboring gene (128). P21 itself is involved in regulation of macrophage activation, septic shock susceptibility (129), autophagy in LPS-induced cardiac dysfunction (130), and cardiomyocyte adheres junctions in endotoxemia (131).

#### TUG1

The lncRNA TUG1 promoted proliferation and migration of VSMCs in the hypertensive state by activating the miR-145-5p/FGF10 axis and the Wnt/β-catenin pathway to aggregate vascular remodeling (132). Another study reported that knockdown of TUG1 ameliorated atherosclerosis via upregulation of miR-133a expression following its target gene FGF1 (133). TUG1 expression was also reported to help alleviate acute lung injuries by targeting miR-34b-5p/GAB1 (134).

### Another Strategy in Searching for lncRNAs

It is theoretically possible to regulate typical molecules of signal pathways by interfering with their corresponding lncRNAs. However, in terms of the extensive functions of those pathways, this train of thought is a low priority. For example, lncRNA HIFa-AS is a natural antisense transcript of Hypoxia-inducible factor 1-α (HIF1α) and is overexpressed in the failing heart. HIFa-AS destabilizes the mRNA producing HIF1α, which regulates transcription of cellular responses to hypoxia, especially in post-ischemic angiogenesis (135). HIFa-AS was also discovered to play a role in MI (98). Huang et al. reported that lncRNAs upregulated in sepsis were significantly enriched in the HIF-1 signaling pathway via KEGG analyses (26), and two studies found that HIF-1α participated in acute lung injury after sepsis (136, 137). Nevertheless, there are no studies reporting the role of lncRNA HIFa-AS in sepsis.

## lncRNAs as Biomarkers and Therapeutic Targets

SICD is more like a functional disorder than a biochemical phenomenon. There is uncertainty as to whether SICD itself is pathogenic or is simply a reflection of the severity of the underlying disease process of sepsis. Diagnosis of SICD largely relies on ultrasonography imaging and troponin measurement. Increasing ultrasonic measurement indicators of left ventricular systolic and diastolic performances, and right ventricular dysfunction are applied to clinical practice and scientific research (4). Similar to troponin, the elevation of hormones B-type natriuretic peptide (BNP) and N-terminal pro-BNP (NT-proBNP) are determined mainly by the severity of sepsis other than specific abnormalities in cardiac function (138). To date, no ultrasonic prognostication has been demonstrated in patients with septic cardiomyopathy (139–142). Apart from lacking reliable biomarkers, the degree to which cardiac dysfunction represents cardiac structure damage and heart failure instead of a protective hibernation-type mechanism remain difficult to resolve (143). Current therapy for sepsis is predominantly focused on restoring cardiac output by inotropic agents and fluid resuscitation. The Surviving Sepsis Campaign guidelines recommend inotropic therapy in patients with persistent hypoperfusion despite adequate fluid loading (144). Limited and underperforming inotropic agent options, including dobutamine, catecholamines, and levosimendan, also contribute to SICD-related deaths (145–147). Therefore, novel biomarkers and therapeutic targets are urgently needed to improve the diagnosis and treatment of SICD.

### LncRNAs as Predictive Biomarkers

As previously discussed, numerous lncRNAs are aberrantly expressed in SICD compared with normal cardiac tissue or cell lines, and this is useful to distinguish SICD patients from healthy cohorts. Although nearly all lncRNAs with direct evidence of their involvement in SICD were declared as potential biomarkers of SICD, those lncRNAs also show aberrant expression patterns in sepsis without cardiac dysfunction, especially critical patients, and in other non-sepsis situations such as MI, I/R, and acute kidney injury (21, 22, 24, 26, 110, 114, 148). This reduces the reliability of using these lncRNAs as potential biomarkers of SICD. To date, there is no study reporting the sensitivity and specificity of the diagnostic efficiency of these lncRNAs.

Compared with myocardial biopsy, blood sampling is largely non-invasive and thus is an ideal diagnostic approach. Several SICD-related lncRNAs can be present in the blood, as are the aforementioned cases. However, the circulating lncRNA differential expression profile is heterogeneous among different studies, partly due to severity, genetic background, and the pathogenic microorganism involved (149). One challenge with the clinical application of these lncRNAs is how to develop a convenient and rapid technique to detect the target lncRNAs in sepsis and thus bring the advantage of being less time-consuming than microbial culture into full play.

### LncRNAs as Therapeutic Targets

To date, many studies have confirmed that lncRNAs are essential contributors to SICD progression due to the diversity of actions and cellular processes implicated. However, few practical examples of therapeutic applications of lncRNAs have been reported. The prognosis of SICD in general is poor, and this is in part due to the lack of therapeutic targets. The critical roles of lncRNAs in SICD make them promising targets for novel therapeutic interventions, and the base-pairing principle is much more straightforward than designing a specific protein-binding inhibitor. Multiple different approaches can be used in perturbing specific lncRNAs, including RNA interference (RNAi), antisense oligonucleotides (ASOs), clustered regularly interspaced short palindromic repeats (CRISPR)/Cas, CRISPR-Display, and the λN –Gal4 system.

Some lncRNAs, which regulate transcriptional outputs in *cis*, do not function in exogenous overexpression studies. Therefore, the λN –Gal4 system has been used to overcome this constraint by enhancing the overexpression of lncRNA in *cis* (150, 151). As duplex RNAs that have to be loaded into AGO2 protein to form an RNA-induced silencing complex (RISC) and interact with target lncRNA, RNAi is a reliable approach for targeting lncRNAs in the cytoplasm and inhibiting gene expression (152). Compared with RNAi, ASOs, as single-stranded DNAs, are more reliable gene silencing agents than duplex RNAs for the RNAs that are localized to cell nuclei (153, 154). ASOs with appropriate modifications have become readily available (155) and newer generation ASOs allow spatial control of target delivery (156). However, unlike RNAi (42, 53), no study to date has reported the application of ASOs in SICD. With higher efficiency, specificity, and the ability to modulate gene expression (157), the CRISPR/Cas method has dominated in recent years (158, 159) and CRISPR Display, which allows the insertion of RNA domains into DNA loci, was specially developed to modulate the expression of lncRNAs (160). Except for cell and animal models, ASOs and RNAi have already been applied in clinical trials for treatment of HBV (161).

## Conclusion

Sepsis-induced cardiac dysfunction is challenged by a lack of uniformity in its definition of incidence, prognosis, and clinical importance. Two other core problems are whether cardiac dysfunction definitely contributes to poor outcome or prognosis, or is simply a reflection of organ failure in general, and the degree to which sepsis-induced cardiac dysfunction is adaptive or pathological (4). Construction of an ideal SICD animal model is difficult and existing research on this condition has only utilized sepsis models to investigate cardiac dysfunction, which partly accounts for the ambiguous mechanisms of SICD. The development and widespread use of GWAS and RNA-Seq has facilitated more discoveries and deeper understanding of lncRNAs, which in turn has helped exploration of SICD regulatory circuits and molecular mechanisms to make a comprehensive and clear definition of this condition, rather than it simply being based on observation of clinical patients.

Several limitations and challenges need to be solved before lncRNAs can reach clinical application. A primary concern is how to specifically target certain tissues or cell populations. As previously mentioned, nearly all identified lncRNAs in SICD or sepsis exhibit functions in other organisms or display multiple mechanisms of action. Second, it is well-established that unlike protein-coding genes, the majority of human long non-coding RNAs (lncRNAs) are considered non-conserved, suggesting variable evolutionary pressure between mRNA and lncRNAs (162). LncRNA conservation includes four dimensions: the sequence, structure, function, and expression from syntenic loci (163). However, several lncRNAs, such as HOTAIR (164) and Xist (165), exhibit clear functional roles in various mammalian species with poor sequence conservation (166). This phenomenon may be due to conserved secondary structures that do not alter with mutations in the sequence outside of structural regions (167, 168). Lnc-H19 and MALAT1 has been proved to be promising targets for cancer therapy (169, 170). Most of published studies of homolog lncRNAs were related to cancers, and now, more than forty clinical trials associated with lncRNA, including a study of lnc-NBR2 in sepsis, are in process in clinicaltrials.gov. Moreover, owing to unique secondary structure, circRNAs resist degradation by exoribonucleases, resulting in more abundant expression. Long-read sequencing technologies promise to improve current annotations and provide a novel perspective to locate homologs in human (171). However, secondary structures are more difficult to intervene in by conventional means than sequence mutation based on existing knowledge and technology. This leads to difficulties in constructing lncRNA knockout animal models. In addition, Joung et al. (172) reported that ~50% of lncRNAs influence the expression of neighboring protein-coding genes and many lncRNAs overlap with protein-coding genes, making it difficult to specifically knockout a lncRNA without affecting neighboring genes. RNA modification, especially m6A modification, also influences lncRNA function, for example in the case of m6A of Xist (173). Furthermore, the finding that micropeptides are encoded by lncRNAs (174) means research on lncRNAs has become more complicated and confusing. Overall, lncRNA of SICD is a promising field and remains largely undiscovered.

## Author Contributions

DZ and YL conceived the presented idea. JL, YZ, and YL summarized the reference and drafted the manuscript. JL drafted the table. DZ organized the figure with online free material. DZ and YL supervised the project and contributed equally to the final version of the manuscript. All authors contributed to the article and approved the submitted version.

## Conflict of Interest

The authors declare that the research was conducted in the absence of any commercial or financial relationships that could be construed as a potential conflict of interest.

## Abbreviations

ASOs: antisense oligonucleotides
CAD: coronary artery heart disease
circRNAs: circular RNAs
CLP: cecal ligation and puncture
CMVECs: cardiac microvascular endothelial cell
CRISPR: clustered regularly interspaced short palindromic repeats
CVD: cardiovascular disease
DAMPs: damage-associated molecular patterns
GWAS: genome-wide analyses
HCAECs: human primary coronary artery endothelial cells
HCASMC: coronary artery smooth muscle cells
HIF1α: Hypoxia-inducible factors 1α
HUVECs: human umbilical vein endothelial cells
I/R: Ischemia/Reperfusion
lncRNAs: long non-coding RNAs
LPS: lipopolysaccharide
MI: myocardial infarction
NF-κB: nuclear factor κB
PAMPs: pathogen-associated molecular patterns
PBMCs: Peripheral blood mononuclear cells
RNAi: RNA interference
SICD: sepsis-induced cardiac dysfunction
XIAP: X-chromosome-linked inhibitor of apoptosis
VSMCs: vascular smooth muscle cells.
